# Elimination of bacterial DNA during RNA isolation from sputum: Bashing bead vortexing is preferable over prolonged DNase treatment

**DOI:** 10.1371/journal.pone.0214609

**Published:** 2019-03-28

**Authors:** Csilla Paska, Imre Barta, Orsolya Drozdovszky, Balazs Antus

**Affiliations:** 1 Department of Pathophysiology, National Koranyi Institute of Pulmonology, Budapest, Hungary; 2 Department of Pulmonology, National Koranyi Institute of Pulmonology, Budapest, Hungary; University of Helsinki, FINLAND

## Abstract

Sputum often contains large amounts of contaminating bacterial DNA that, if not eliminated during RNA isolation, may interfere with gene expression studies. During RNA isolation only repeated DNase treatment can effectively remove contaminating bacterial DNA from samples, but this compromises RNA quality. In this study we tested alternative methods to facilitate the removal of DNA and improve the quality of RNA obtained. Sputum samples obtained from patients with chronic obstructive pulmonary disease were processed with dithiothreitol and subjected to various RNA isolation methods, yet with modified protocols. Modifications included prolonged DNase treatment or vortexing of sputum cells in the presence of beads prior to RNA isolation. Bacterial DNA contamination was tested by PCR using universal bacterial primers, while RNA quality was assessed by real-time PCR using GAPDH primers for amplicons of different length. We found that the RNeasy Plus Mini kit equipped with the gDNA eliminator spin column was able to completely eliminate bacterial DNA, if sputum cells were lysed in the presence of bashing beads. Notably, compared with the standard protocol, the modified procedure yielded better quality RNA as well, as indicated by improved threshold profiles of qPCR. Bead vortexing of cells was less effective when combined with other RNA isolation methods, and the repeated DNase treatment needed to completely remove contaminating DNA from the samples reduced the quality of RNA markedly. Bead vortexing in combination with certain RNA extraction methods greatly facilitates the isolation of sputum RNA that is free of contaminating bacterial DNA, and is suitable for downstream applications.

## Introduction

Sputum is a valuable source of cells, proteins, lipids, nucleic acids and oxidative products, and it is an ideal sample for studying lung physiology [1–4]. Assessment of these molecules and processes may have clinical implications as well; for example, gene expression profiling of sputum samples may identify distinct phenotypes among patients with asthma [5] or chronic obstructive pulmonary disease (COPD) [6], and could also be a promising screening tool for detecting lung cancer [7]. Of importance, sputum as a clinical sample is often preferable over blood, as it is lung-specific, while blood contains a mixture of signature markers of several pathophysiological processes unfolding simultaneously in a patient [8].

Despite the advantages, relatively few eukaryotic gene expression studies from sputum have been published to date, partly due to the challenges involved in the isolation of sputum RNA. Sputum samples are often difficult to obtain, need to go through a lengthy processing protocol, and therefore generally yield RNA of low quantity and quality with respect to RNA isolated from other biological samples. The ubiquitous presence of bacteria in sputum samples aggravates the problems even further.

Previously, we have developed a combined sputum processing/RNA extraction/reverse transcription-quantitative polymerase chain reaction (RT-qPCR) protocol to maximize the quantity and the quality of sputum RNA in order to enhance detection of transcripts by qPCR [9]. The elimination of contaminating bacterial DNA during RNA extraction proved to be the biggest challenge in the development of that protocol. Several rounds of DNase treatment was required that not only led to a 30–50%, reduction of initial nucleic acid content but negatively affected RNA quality as well.

In this study we aimed to improve the efficiency of eliminating contaminating DNA without affecting RNA quality. Tested modifications to common RNA isolation procedures included prolonged DNase treatment and bead vortexing of sputum cell fractions during cell lysis, a recently developed technique for disrupting tough-to-lyse bacteria in biological samples.

## Materials and methods

### Sputum collection and processing

Induced sputum samples were collected from 10 clinically stable patients with chronic obstructive pulmonary disease (COPD) (mean age: 65.3±4.4 years, GOLD II-IV. stages) during routine ambulatory visits. Sputum induction and processing with dithiothreitol (DTT) was performed as previously described [10,11]. Briefly, sputum samples were homogenized in PBS containing 0.1% DTT and filtered through a 40 μm mesh and centrifuged. An adequate sample was defined if the number of squamous epithelial cells was less than 20% of the total number of cells in the sputum. Cytospins were then prepared and stained with May-Grunwald-Giemsa for differential cell counting. At least 400 inflammatory cells were counted for each slide. The various inflammatory cells in sputum were shown as a percentage of total viable non-squamous cells. Finally, samples were aliquoted to 10^6^ inflammatory cells per cryotube. From each sputum sample as many aliquots were prepared as possible. Aliquots were centrifuged and finally the pellet was re-suspended in 150 μL RNA*later* solution (Sigma-Aldrich, St. Louis, MO, USA) and stored at -80°C. The research protocol was approved by the Hungarian Scientific and Research Ethics Committee of the Medical Research Council (ETT TUKEB, No: 43842-4/2018/EKU), and all subjects gave written informed consent to participation in the study.

### RNA isolation and bead treatment

RNA isolation was performed from 10^6^ sputum cells using either (*i*) Trizol Reagent (Life Technologies, Foster City, CA), (*ii*) NucleoSpin TriPep kit (Macherey-Nagel, Düren, Germany), (*iii*) Direct-zol RNA MiniPrep kit (Zymo Research, Irvine, CA) or (*iv*) RNeasy Plus Mini kit (Qiagen, Düsseldorf, Germany) as previously described [9] with the modification that prior to RNA isolation, sputum cell fractions were vortexed for 5 min in the presence of BashingBeads (mixed 0.5 mm & 1.0 mm; Zymo Research, Irvine, CA) or glass beads (3 mm). Bead vortexing took place in the respective proprietary lysis buffer of each kit. Following centrifugation at 12000 *g* for 1 min samples were loaded onto the corresponding RNA isolation column.

Integrity of the isolated sputum RNA was estimated using the cycle threshold (Ct) values of different base pair (bp)-length amplicons of glyceraldehyde 3-phosphate dehydrogenase (GAPDH) in qPCR assays as previously described [12].

### DNase digestion

DNase digestion (1 hour and 24 hours) was performed after RNA isolation using the Turbo DNA-free kit (Life Technologies, Carlsbad, CA, USA).

### PCR

The presence of contaminating bacterial DNA was checked by PCR using universal bacterial primers producing a 466 bp long amplicon [13,14**]**. To check for contaminating human genomic DNA, GAPDH primers were used, which give a 121 bp long PCR product for cDNA and a 210 bp product for genomic DNA [12**]**. PCR was performed with Phire DNA Polymerase kit (Thermo Fisher Scientific, Waltham, MA, USA) as described previously [9**]**. In pilot experiments alternative DNA polymerases, such as the 5PRIME HotMaster Taq DNA Polymerase kit (5Prime GMBH, Hilden, Germany), the Phusion DNA (Thermo Fisher Scientific) and the Pfu DNA Polymerase kits (Thermo Fisher Scientific) were also tested.

Amplicons were identified by electrophoresis (100V, 60 min) on 2% agarose gel (Serva, Heidelberg, Germany) cast in TBE buffer (Duchefa Biochemie, Haarlem, Netherlands). For visualization 7 μL GR Safe nucleic acid stain (Lab Supply Mall, Gaithersburg, MD, USA) was used.

### RT

0.5 μg of total RNA in 20 μL reaction volume was reverse transcribed using the High Capacity cDNA Reverse Transcription kit (Life Technologies) as previously described [9]. Random hexamers (250 nmol) were used as primers.

### Real-time PCR

Real-time PCR (qPCR) reactions were carried out with 2 μL cDNA sample in a total reaction mixture of 20 μL containing either 10 μL 2× iTaq Universal SYBR Green Supermix (Bio-Rad, Hercules, CA, USA) and 250 nmol each of GAPDH forward and reverse primers giving rise to 75, 121, 225 and 406 bp long amplicons (9) or 10 μL 2× iTaq Universal Probes Supermix (Bio-Rad) and 1 μL GNB2L1 Taqman probe (Life Technologies Hs00914568_g1) as reference gene. Amplification in qPCR assays was performed as previously described [9].

### Statistical analysis

Data are presented as mean±standard error of mean (SEM). Data distribution was analyzed by the Kolmogorov-Smirnov test. Ct values were compared using one-way analysis of variance with Newman-Keuls test for multiple comparisons. Comparisons between two groups were performed by the Student’s *t*-test. Calculations were performed by GraphPad Prism 4.0 (GraphPad Software Inc., San Diego, CA, USA). A p value <0.05 was considered significant.

## Results

### Selecting a suitable DNA polymerase

An integral part of the study protocol was to check for the presence of contaminating bacterial DNA in the isolated sputum RNA. PCR using universal bacterial primers was the method of choice and for this to be informative we had to make sure that no exogenous bacterial DNA was introduced during cDNA synthesis. Thus, a suitable DNA polymerase that was free from contaminating bacterial DNA had to be selected first. In pilot PCR experiments using a complete reaction mix but no RNA template it was found that the 5PRIME and the Phusion polymerases are contaminated with bacterial DNA, while the Phire and the Pfu kits presented no such problems (S1 Fig). The Phire DNA polymerase was selected for all subsequent experiments.

### Effect of prolonged DNase treatment on the quality of sputum RNA

**Fig 1:**
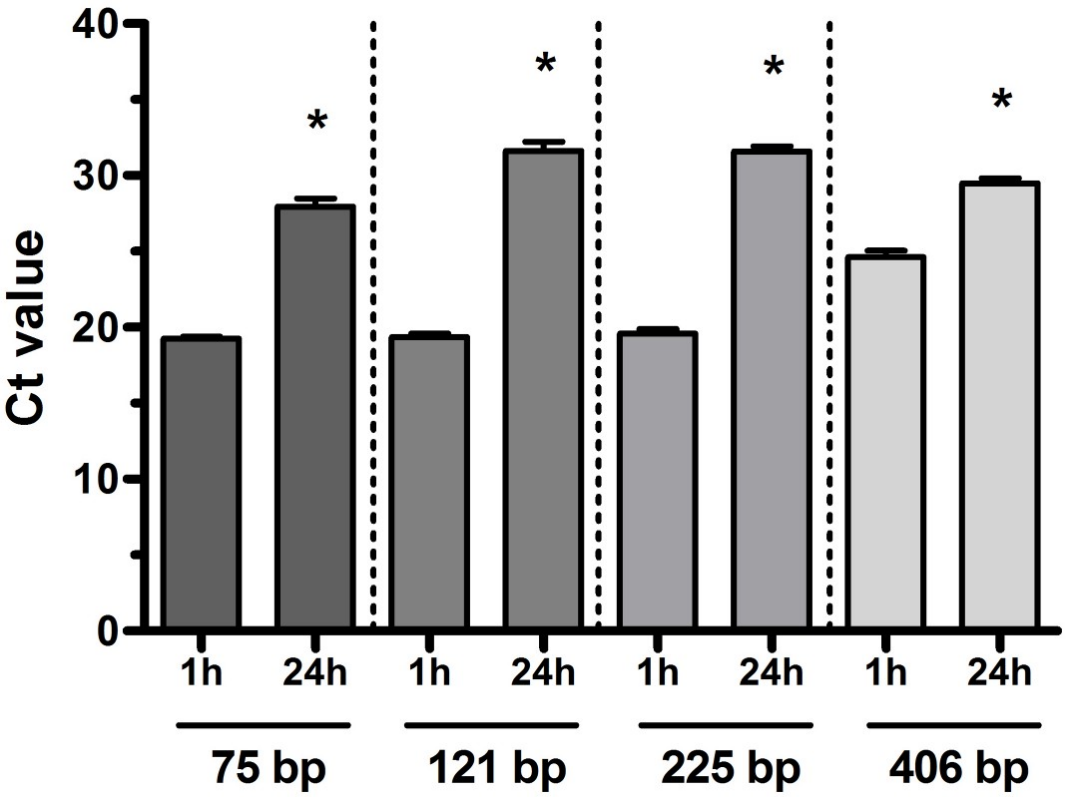
Effect of different DNase incubation times (1 and 24 h) on cycle threshold (Ct) values for real-time PCR assays using different base pair (bp) long amplicons of glyceraldehyde 3-phophate dehydrogenase. Each column represents the average of 6 separate reactions. Error bars indicate SEM. *p<0.05 1 hour vs. 24 hours of DNase treatment.

As demonstrated previously, strictly following the manufacturer’s recommendations, none of the RNA isolating methods tested was capable to yield DNA-free RNA from sputum as the recommended 1-hour long DNase treatment proved insufficient to eliminate contaminating bacterial DNA from the samples [9]. Prolonged DNase treatment (up to 24 hours) removed more contaminating DNA, but a concomitant increase in RNA degradation occurred, as evidenced by increased Ct numbers in RT-qPCR (Fig 1).

### Effect of bead vortexing on contaminating bacterial DNA

**Fig 2:**
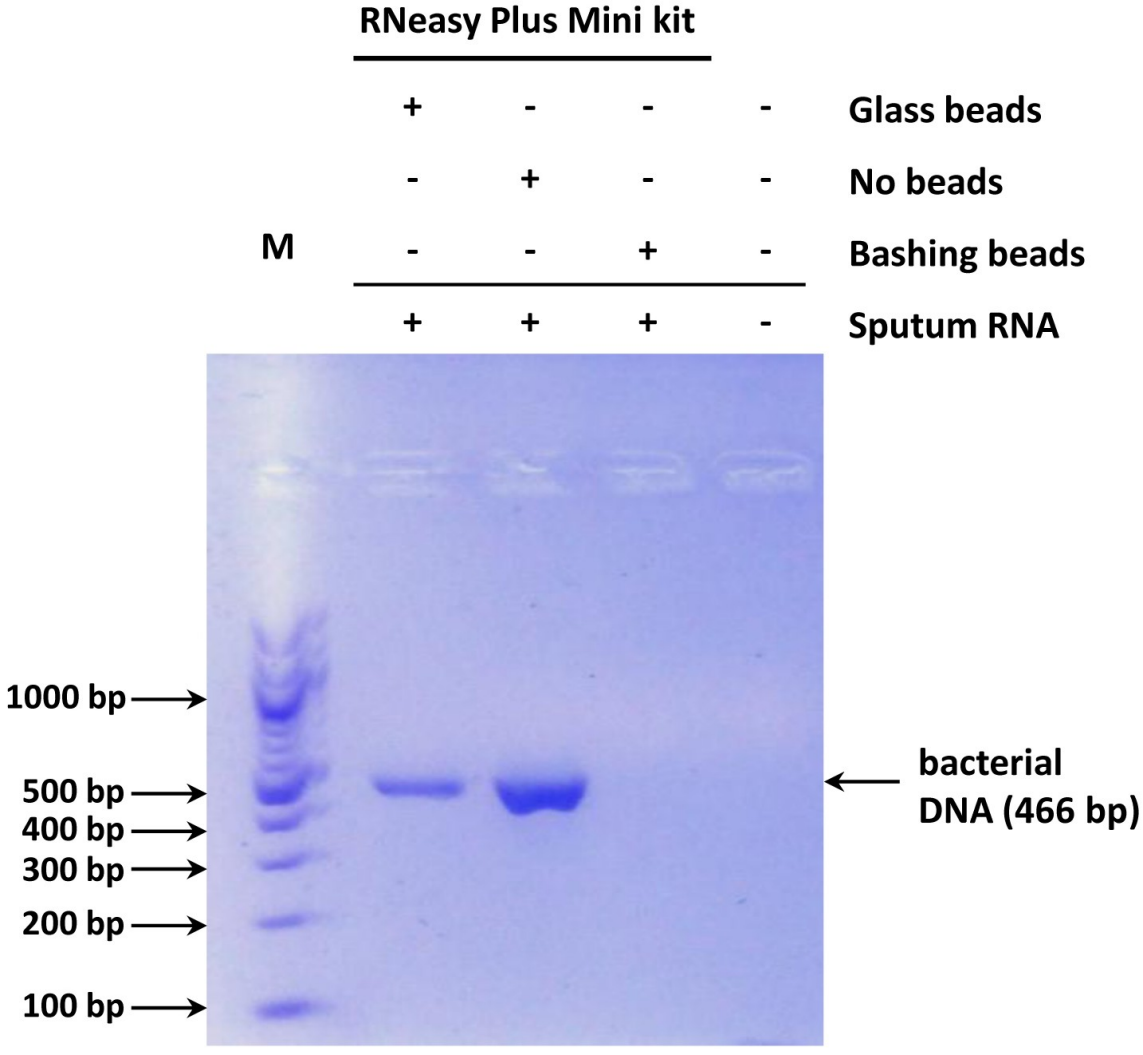
Representative gel electrophoresis of PCR reactions using universal bacterial primers and sputum RNA isolated with the RNeasy Plus Mini kit. Sputum cells were either subjected or not to bead vortexing (bashing or glass beads) *prior* to RNA isolation. Bands representing contaminating bacterial DNA are indicated. M: molecular weight marker.

**Fig 3:**
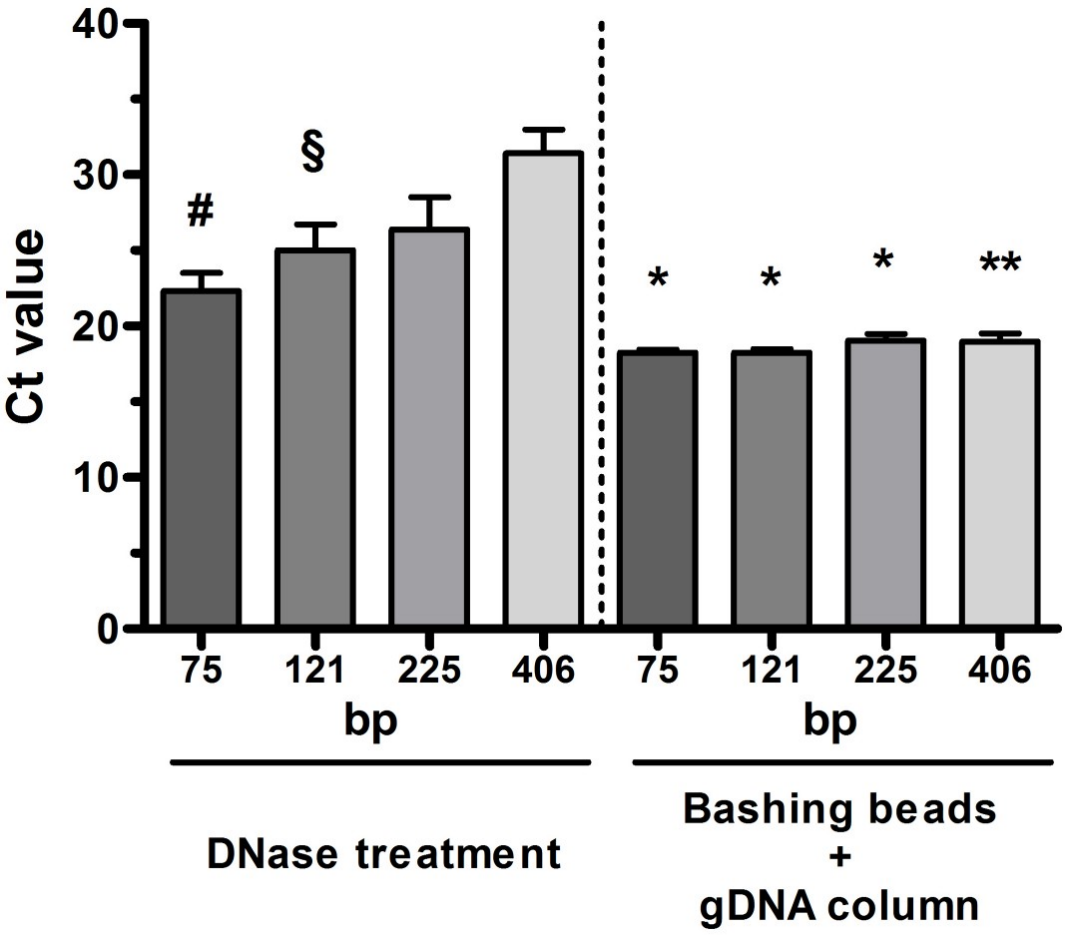
Cycle threshold (Ct) values for real-time PCR assays with different base pair (bp) long amplicons of glyceraldehyde 3-phophate dehydrogenase on RNA isolated from sputum samples. Samples were subjected either to repeated digestion process (up to 6 times) with the Turbo DNA-free kit or bead vortexing *prior* to RNA isolation using the RNeasy Plus Mini kit equipped with the gDNA eliminator spin column (n = 6 for each group). Error bars indicate SEM. ^#^p<0.01 and ^§^p<0.05 vs. 406 bp long amlicons, *p<0.005 and **p<0.001 vs. DNase-treated samples.

Next, we tested whether vortexing in the presence of various beads during the cell lysis step of RNA isolation protocols could facilitate the removal of bacterial DNA. We found that vortexing sputum cells with bashing beads and subsequent loading of the lysate onto the gDNA eliminator spin column of the RNeasy Plus Mini kit was necessary and sufficient to completely eliminate DNA from the samples (Fig 2). Notably, bead vortexing also improved the threshold profiles of qPCR indicating that better quality RNA was obtained (Fig 3).

Bead vortexing was less effective in combination with other RNA isolation methods (Trizol, Tripep and Direct-Zol, S1 Table). In each case several rounds of DNase treatment was additionally necessary to eliminate contaminating bacterial DNA from the samples.

Simultaneously, glass beads were also tested. We found that vortexing sputum cells in the presence of glass beads helped eliminating more contaminating DNA with respect to the original protocols, but it was not as effective as bashing beads, and some DNA always remained in these samples (Fig 2, S1 Table).

### Optimizing RNA isolation using the RNeasy Plus Mini kit

The RNeasy Plus Mini kit offers two alternative protocols; the standard protocol provides enrichment of the sample with intact mRNAs by eliminating RNAs shorter than 200 nucleotides; the second protocol is designed for the purification of total RNA. The latter might be preferable for studies involving samples such as sputum where the whole spectrum of RNAs, even partially degraded ones, are to be detected. When comparing the qPCR profile of the two protocols we found that the total RNA protocol yields more short amplicons, while the amount of long amplicons were comparable (S2 Fig).

## Discussion

Sputum is a valuable source for molecular analysis of respiratory diseases. The suitability of sputum for gene expression studies, however, is often hindered by the ubiquitous presence of bacteria. As demonstrated in our previous study, several commercially available RNA isolation kits fail at yielding DNA-free RNA from sputum [9]. To circumvent the problem, we had proposed modifications to the RNA isolation protocol that among other optimization steps, involved repeated cycles of DNase digestion. Although beneficial for removing contaminating DNA, this modification was suboptimal in the sense that it was not only time consuming, but compromised the integrity of the extracted RNA as well.

In the current study we tested alternative approaches for obtaining DNA-free RNA from sputum. We demonstrated that mechanical disruption of bacterial cell walls, i.e. vortexing sputum cells in the presence of small, chemically inert ceramic beads (bashing beads) allows the efficient and complete elimination of bacterial DNA from samples using the RNeasy Plus Mini kit protocol. Bead bashing obviates the need for repeated or prolonged DNase digestion, and, as a consequence, RNA of better quality can be obtained, as evidenced by the improved threshold values for both short and long amplicons in qPCR assays.

Although most RNA isolation protocols incorporate steps for eliminating DNA, those measures, if at all, only work for DNA already in solution. Certain bacteria are resistant to lysis buffers, therefore DNA within intact bacterial cells can escape measures implemented against DNA contamination during RNA isolation. Cells that withstand the cell lysis step could be a potent source for contaminating DNA during downstream applications that involve boiling samples, such as PCR [15,16]. The presence of significant amounts of contaminating DNA in an RNA sample could impair RT-qPCR assays in several ways. It can lead to an underestimation of the amount of input RNA, and exhausts primer and deoxynucleotide triphosphate pools when random hexamers are used for reverse transcription.

Efficient disruption of bacterial cells before RNA isolation may be a plausible explanation why the use of the gDNA eliminator column alone was insufficient, while in combination with bashing beads treatment it was sufficient for the removal of bacterial DNA from sputum samples using the RNeasy Plus Mini kit. This, however, does not explain why RNA isolated using either the Tripep or the Direct-Zol kits still contained significant amounts of DNA, despite pretreatment of samples with beads. These methods employ solid phase columns for DNA removal as well, but in contrast to the gDNA eliminator column in the RNeasy Plus Mini kit, those columns are incapable of completely eliminating DNA, that is why their respective protocols include an additional DNase step to work efficiently. Thus, it appears that the gDNA eliminator column uses some additional proprietary feature to eliminate the entire DNA in solution. Another plausible explanation for the behavior of these kits was mentioned by Korfhage et al., who demonstrated that highly fragmented nucleic acids often yield high levels of residual gDNA, because the smaller sized fragmented gDNA co-purifies with RNA [17]. Taken together, most commercially available RNA isolation kits and their respective lysis buffers are severely limited for purposes that target bacterially contaminated and/or degraded samples.

Testing DNA content left in the isolates we found that certain commercially available DNA polymerases are contaminated with DNA, confirming earlier observations [18,19]. It is imperative to use certified DNA-free polymerases to avoid artifacts in downstream applications.

It is well known that using low-quality RNA may strongly compromise results of qPCR [20]. The RNA Integrity Number (RIN) would not be informative enough to assess the quality of human mRNA in sputum samples as bacterial RNA is no selectively eliminated using any of the applied RNA isolation protocols. Instead, we assessed the degree of human RNA degradation by looking at the amplification efficiency of different length amplicons of the same housekeeping gene (GAPDH) was assessed [12]. We found that Ct values of longer amplicons (406 bp) were increased compared to those of shorter amplicons (75 and 121 bp) in DNase-treated but not in bead-vortexed samples, indicating that RNA in these latter samples remained more intact. This is reasonable, since the extracted RNA was not exposed to the highly demanding conditions of the digestion process.

Although bacterial cell walls can be disrupted by various treatments including enzymes, detergents and even sonication [21], bead beating is likely a more efficient technique for this purpose [22,23]. The diameter of the beads used for the physical disruption has some impact on the efficiency of the lysis. Our results show that the larger glass beads, compared to the smaller bashing beads, are less effective in removing contaminating DNA, in line with the findings of de Boer et al. on Gram-positive microorganisms [23]. More recently, a magnetic beads-based DNA and RNA co-extraction method for sputum has also been described by He et al. [24]. However, this protocol was developed for the detection of respiratory viruses (both RNA and DNA viruses) in the sputum of children with suspected acute respiratory infections and not for the detection of human gene expression. Therefore, concerns regarding the elimination of bacterial DNA from the samples were not addressed.

Previously we demonstrated that by adapting the conditions of isolation to the special requirements of sputum, the quantity RNA can be greatly increased. In our present work we could show, that instead of repeated DNAse digestion, vortexing sputum cells in the presence of bashing beads improves the quality of sputum RNA, making it compatible with gene expression studies. Our results highlight the importance of selecting a suitable DNA polymerase for cDNA synthesis. We speculate that our findings can be applied to other types of respiratory samples, for example, bronchoalveolar lavage. Just like sputum, lavage is also a rich source of cells and macromolecules, but it may also contain bacteria whose removal poses a similar challenge during RNA isolation.

## Conclusions

Obtaining appropriate amounts of high-quality RNA is a critical step in all gene expression studies, including the ones that utilize sputum. Although in our previous study [9] we already optimized conditions for sputum processing and RT-qPCR assays, the problems associated with the elimination of contaminating bacterial DNA during RNA isolation could not be adequately overcome. Our current study demonstrated that vortexing of cells with bashing beads in combination with a column-based RNA extraction method (RNeasy Plus Mini kit) allows complete removal of the DNA from sputum samples, and provides the best quality RNA. Since this methodological innovation is simple, it can be recommended for everyone who is planning gene-expression studies in sputum.

## Supporting information

S1 Fig Representative gel electrophoresis of PCR control reactions (no RNA samples added) using different DNA polymerases (Phire, 5PRIME, Phusion and Pfu) and universal bacterial primers. The presence of contaminating bacterial DNA in the first two reactions is evidenced by the respective PCR product in lane 2 and 3. M: molecular weight marker.
(TIF)

S2 Fig Cycle threshold (Ct) values for real-time PCR assays using different base pair (bp) long amplicons of glyceraldehyde 3-phophate dehydrogenase on either the total RNA or RNAs longer than 200 bp isolated from the sputum with the RNeasy Plus Mini kit. Error bars indicate SEM.
(TIF)

S1 File Data from the study. (XLSX)

S1 Table Effect of bashing and glass bead vortexing on the bacterial DNA content of sputum samples subjected to different RNA isolation methods/kits (TriPep, Trizol, Direct-zol and RNeasy Plus). The amount of remaining bacterial DNA was visualized by gel electrophoresis; the semi-quantitative results are indicated (DNase treatment was omitted in each case).
(PDF)
