# Supplementary figures and images for: Elimination of bacterial DNA during RNA isolation from sputum: Bashing bead vortexing is preferable over prolonged DNase treatment
Source: PLoS One. 2019 Mar 28;14(3):e0214609. doi: 10.1371/journal.pone.0214609 (PMC6438495; doi:10.1371/journal.pone.0214609)

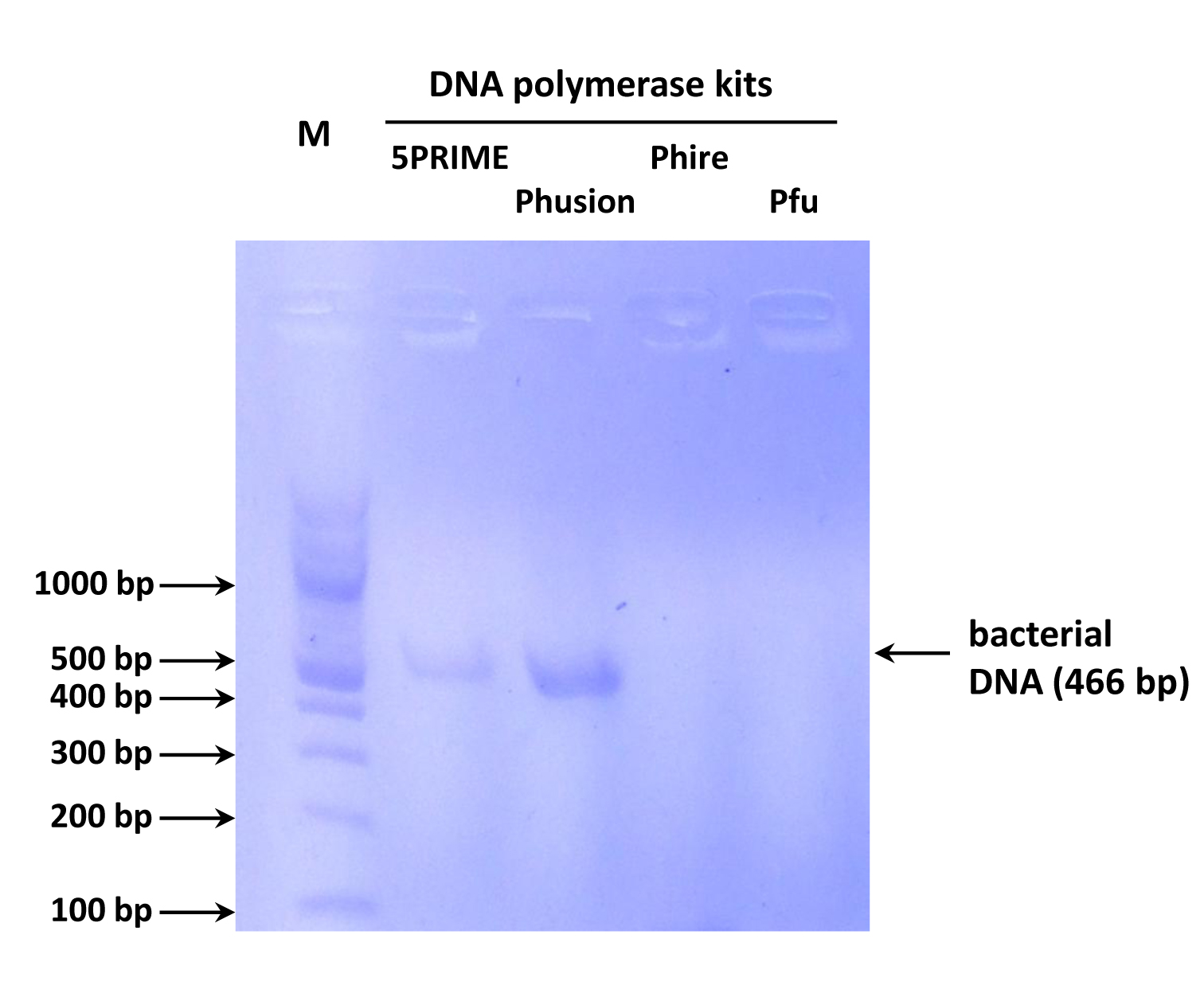

Supplement: S1 Fig — The presence of contaminating bacterial DNA in the first two reactions is evidenced by the respective PCR product in lane 2 and 3. M: molecular weight marker. (TIF) [file pone.0214609.s001.tif]

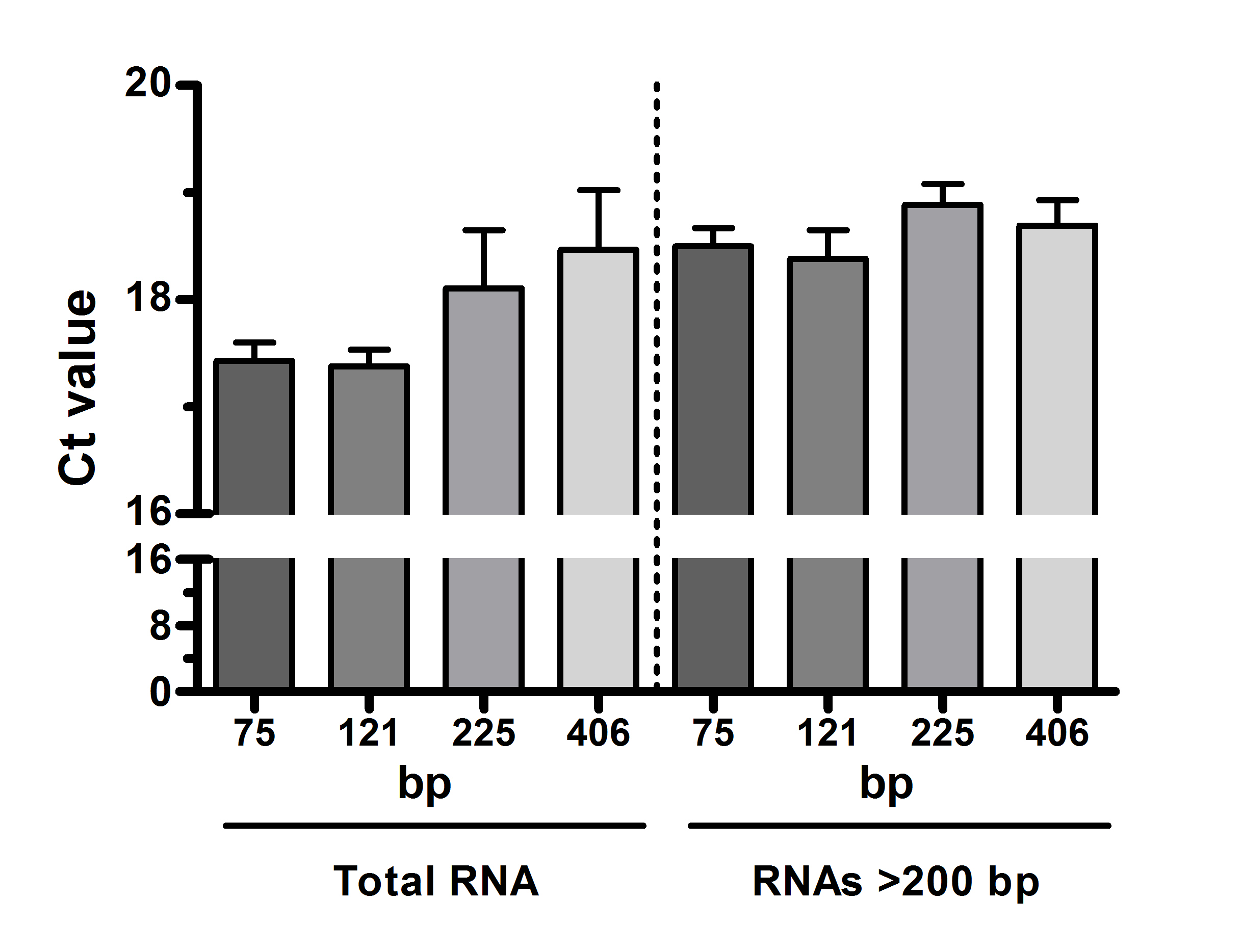

Supplement: S2 Fig — Error bars indicate SEM. (TIF) [file pone.0214609.s002.tif]
