# Supplementary material for: Elimination of bacterial DNA during RNA isolation from sputum: Bashing bead vortexing is preferable over prolonged DNase treatment
Source: PLoS One. 2019 Mar 28;14(3):e0214609. doi: 10.1371/journal.pone.0214609 (PMC6438495; doi:10.1371/journal.pone.0214609)
Supplement: S1 Table — The amount of remaining bacterial DNA was visualized by gel electrophoresis; the semi-quantitative results are indicated (DNase treatment was omitted in each case). (PDF) [file pone.0214609.s004.pdf]

RNA isolation kits	No beads	Glass beads	Bashing beads
TriPep	****	***	*
Trizol	***	**	*
Direct-zol	***	**	**
RNeasy Plus	***	**	None
